# Human-driven breakdown of predator–prey interactions in the northern Adriatic Sea

**DOI:** 10.1098/rspb.2024.1303

**Published:** 2024-09-25

**Authors:** Martin Zuschin, Rafał Nawrot, Markus Dengg, Ivo Gallmetzer, Alexandra Haselmair, Michał Kowalewski, Daniele Scarponi, Sandra Wurzer, Adam Tomašových

**Affiliations:** ^1^ Department of Palaeontology, University of Vienna, Josef-Holaubek-Platz 2, Vienna 1090, Austria; ^2^ Otago Regional Council, Dunedin 9010, New Zealand; ^3^ Third Zoological Department, Natural History Museum Vienna, Burgring 7, Vienna 1010, Austria; ^4^ Florida Museum of Natural History, University of Florida, 1659 Museum Road, Gainesville, 32611 FL, USA; ^5^ Department of Biological, Geological and Environmental Sciences, University of Bologna, Bologna, Italy; ^6^ Earth Science Institute, Slovak Academy of Sciences, Dúbravska cesta 9, 84005 Bratislava, Slovakia

**Keywords:** conservation palaeobiology, drilling predation, eutrophication, hypoxia, molluscs, Mediterranean Sea

## Abstract

Long-term baseline data that allow tracking how predator–prey interactions have responded to intensifying human impacts are often lacking. Here, we assess temporal changes in benthic community composition and interactions between drilling predatory gastropods and their molluscan prey using the Holocene fossil record of the shallow northern Adriatic Sea, which is characterized by a long history of human transformation. Molluscan assemblages differ between the Isonzo and Po prodelta, but both show consistent temporal trends in the abundance of dominant species. Samples of mollusc prey collected at high stratigraphic resolution indicate that drilling frequencies have drastically declined in the Po prodelta since the mid-twentieth century, while a weaker trend in the more condensed sediments of the Isonzo prodelta is not statistically significant. The decrease in drilling predation intensity and the community turnover are linked to the loss of predatory gastropods and the increased relative abundance of less-preferred prey during the most recent decades. Our results align with data showing the substantial depletion of marine resources at higher trophic levels in the region and indicate that the strong simplification of the food web initiated in the late nineteenth century accelerated further since the mid-twentieth century.

## 1. Introduction

Global warming, ocean acidification, eutrophication and direct human interventions in marine ecosystems such as fishing, bottom trawling and species introduction markedly change ecosystem functioning and influence biotic interactions [1–3]. The removal of top vertebrate predators due to overfishing results in the loss of top-down control [4,5] and marine invertebrates at lower trophic levels often profit from this predator release [6–8]. However, for benthic invertebrates the effect of anthropogenic impacts on predator–prey interactions at lower trophic levels is poorly known over decadal to centennial timescales. This gap is driven by the lack of long-term time series pre-dating the onset of anthropogenic disturbances [9,10]. However, ichnological signatures of interspecific interactions preserved in the youngest fossil record (i.e. traces of predation) can be used to reconstruct recent shifts in predation pressure and to place those shifts in a broader historical context [9–13]. In particular, drill holes produced by gastropods in shells of other molluscs provide direct evidence of predation [14] and are one of the most widely used sources of data on predator–prey interactions in the fossil record [15,16], accounting for as much as 75% of predation occurrences documented in the palaeontological literature [17].

**Figure 1.**
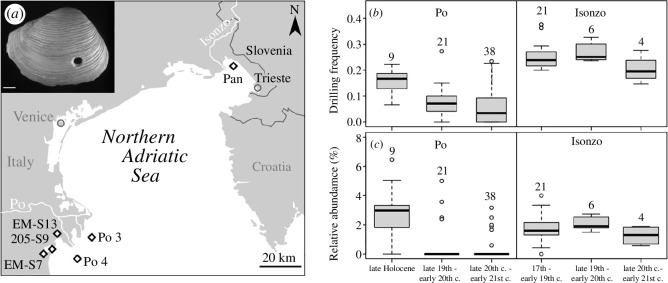
(*a*) Location of the sampling sites in the NAS and on the Po plain near Ferrara. Pan = Panzano. The inset shows the right valve of *V. gibba* with a predatory drill hole. The scale bar on inset represents 1 mm. (*b*) Median drilling frequencies (DFs) of the total assemblage and (*c*) median relative abundance of predators in time bins in both regions.

Here, we use the frequency of predatory traces preserved in the sedimentary record of the northern Adriatic Sea (NAS; figure 1*a*) to reconstruct changes in the frequency of predatory attacks of shell-drilling gastropods on their molluscan prey since the late nineteenth century and to assess the magnitude of those changes relative to the late Holocene baseline pre-dating the onset of intense anthropogenic activities. Over the last 10 000 years, the intensity of drilling predation increased across the basin in step with the increase in organic enrichment and water depth caused by the Holocene transgression [18]. However, the NAS also ranks among the most strongly altered marine regions worldwide, with anthropogenic impact on coastal ecosystems reaching back beyond Roman times and rapidly intensifying over the last century [19–21]. Since the mid-twentieth century, the benthic community composition has changed over the entire northern Adriatic basin [22–25] in response to persistent anthropogenic pressures, including bottom trawling, eutrophication and hypoxia [26–30]. Molluscan communities were subjected to a basin-wide loss of epifaunal species, a reduction of grazers, carnivores and herbivores, and a concurrent increase in the dominance of infaunal and opportunistic species feeding on plankton or detritus [31]. A case in point is the opportunistic, infaunal, filter-feeding bivalve *Varicorbula gibba*, which increased in abundance and body size during the last decades [32]. This regional-scale population shift, unprecedented relative to the Holocene history of this species, was hypothesized to be driven by ecological release from predation, competition and trophic amensalism [25].

Here, we quantify changes in the composition of the molluscan fauna and drilling frequencies (DFs) in sediment cores to document temporal trends in predator–prey interactions between shell-drilling gastropods and their molluscan prey across the Holocene–Anthropocene transition in the NAS. Specifically, we evaluate the impact of human-induced loss of predatory gastropods and other community-level compositional changes on predator–prey interactions.

## 2. Material and methods

Sediment cores were collected at three stations in the NAS in 2013, with two stations located in the Po prodelta at 21 m water depth (Po 3, Po 4), and one station in the Isonzo prodelta in the Gulf of Trieste at 12 m water depth (Panzano) (figure 1*a*, electronic supplementary material, table S1). These two deltaic systems are characterized by the highest sediment accumulation rates in the northern Adriatic Sea [33,34] and thus provide high, decadal-scale resolution of stratigraphic archives [25,35]. At all three stations, the seafloor is muddy. At the Po sites, mud mounds generated by the thalassinid shrimp *Jaxea nocturna* and by the polychaete *Sabella pavonina* are abundant [35]. At Panzano, epifaunal clumps formed by sponges, ascidians, ophiurids, polychaetes and bivalves dominate [36]. Changes in community composition and DF were assessed in two 16 cm diameter replicate cores at each station [37], which were taken a few metres from each other (electronic supplementary material, table S1). These replicate cores are very similar in faunal composition [35,38] and were pooled in this study to increase sample size per increment when analysing DFs and community composition. The cores were sliced into 2 cm increments for the uppermost 20 cm, and 5 cm increments below. The smaller increments at the top were implemented to potentially resolve more finely the surface strata, which record the time interval during which anthropogenic impacts intensified. However, for the analyses presented here, the adjacent 2 cm increments were pooled to increase sample size for individual time intervals and obtain a better comparability with the 5 cm increments used for the rest of the core [35,38].

The core age models are based on radiocarbon-calibrated amino acid racemization of shells of *V. gibba* from cores M13 and M21 in the Po delta [35] and core M28 in the Isonzo prodelta [38]. Time averaging of molluscan assemblages in individual increments varies between 10 and 50 years at the Po prodelta and between approximately 20 years and a few centuries at the Isonzo prodelta (figure 6 in Ref. [35]). Four distinct temporal units were distinguished using the age models based on *V. gibba*, including the middle to late Holocene with 40 samples, the seventeenth to early nineteenth century with 21 samples, the late nineteenth to early twentieth century with 32 samples and the late twentieth to twenty-first century, with 42 samples.

Sedimentation rates at the Panzano station are approximately 2–4 mm/yr and the sedimentary record of the cores covers approximately the last 500 years of ecosystem history [39], providing baseline data on variability in DF and community composition before the onset of strong human impacts in the twentieth century. Conversely, the sediment cores from the Po prodelta provide a higher stratigraphic resolution but extend back only to the late nineteenth century due to high sedimentation rates of approximately 10–20 mm/yr [35]. Therefore, we included older samples from three sediment cores (205-S9, EM-S7 and EM-S13) drilled on the coastal Po plain near Ferrara to evaluate the significance of the recent ecological changes in the context of longer temporal trends. These older samples represent middle to late Holocene prodelta sediments (1.1 to 7.0 ky BP [40]), deposited under similar environmental conditions but subsequently buried under the prograding delta front and coastal plain deposits, and thus currently located at 19 to 26 m below the surface of the present-day Po plain.

All samples were sieved using a 1 mm mesh size and only complete molluscan shells and valves (>90% preserved) were counted and identified to the species level. The abundance of bivalves was calculated as the number of articulated specimens plus the combined number of left and right valves divided by two. To estimate DF (i.e. the per cent of prey specimens with complete drill holes representing a proxy for the frequency of successful predatory attacks), the total number of drilled shells was divided by the total number of bivalve, gastropod and scaphopod individuals per core increment. Because only one valve in bivalves is usually drilled, DF was corrected using the equation of [41]: DF = DV/[(RV + LV)/2 + A], where DV is the total number of drilled valves, RV is the total number of right valves, LV the total number of left valves and A the number of articulated individuals. Only samples with at least 20 individuals were used in the analyses. In addition to the DF of the total assemblage we also calculated DFs for bivalves, gastropods and the four most abundant species, including the bivalves *V. gibba* and *Kurtiella bidentata* and the gastropods *Turritellinella tricarinata* and *Tritia varicosa* (electronic supplementary material, figure S1, table S2). The four species, however, are not abundant enough in temporal bins to make a rigorous comparison of DFs within and between cores at the species level (electronic supplementary material, table S3). Whereas high-resolution analyses of DFs are not possible for individual taxa, we supplemented assemblage-level analyses with coarse-resolution assessments of the four common species. In that case, core samples were pooled into pre-anthropogenic (middle-late Holocene, seventeenth to early nineteenth century, late nineteenth to early twentieth century) and post-anthropogenic (late twentieth to early twenty-first century) and Fisher’s test was applied to assess for statistical differences between the two sample groups. Predatory gastropods, which make up only 1.38% of the total molluscan assemblage, are strongly dominated by two species of naticids, *Euspira nitida* (70%) and *E. macilenta* (20%), while the few muricids (9%) are mostly juveniles of *Hexaplex trunculus* and *Bolinus brandaris.* We were not able to distinguish consistently between drill holes made by these two families of shell-drilling gastropods.

We evaluated stratigraphic changes in the composition of molluscan assemblages using non-metric multidimensional scaling ordination (NMDS; *k* = 2 dimensions) based on square-root-transformed proportional species abundances and Bray–Curtis dissimilarities. The differences in molluscan community composition between the four temporal units were tested with non-parametric permutational multivariate analysis of variance (PERMANOVA [42]). Differences in DFs between the units were assessed with the Kruskal–Wallis test and the pairwise Wilcoxon rank-sum test, using *a posteriori* Bonferroni correction for multiple comparisons. Distance-based redundancy analysis was used to assess whether differences in species-level community composition are related to differences in DF [43]. The principal coordinate analysis using Bray–Curtis dissimilarity was performed on the square-root-transformed proportional species abundances, and the resulting ordination scores were subjected to redundancy analysis, using the function ‘capscale’ in the vegan R package [44].

To assess stratigraphic trends in the relative abundance of drilling predators, a rank correlation between the relative abundance of predators and depth in the core was compared with a resampling distribution of Spearman rho values under a null model in which the relative abundance of predators is constant along the core. The resampling distribution is based on a Monte Carlo simulation of vertical trends generated by random drawing of drilling predators with probability given by their overall relative frequency in cores. At each core level, the total number of simulated specimens was based on the actual sample size for this level. For each simulation run, a Spearman rho value was calculated for random data. Positive rho values indicate an upward decline and negative values indicate an upward increase in predator abundance towards the top of the core. The two-tailed significance value *p* is based on the number of replicate runs in which the simulated absolute values of rho exceeded the observed absolute rho value. The results are based on 10 000 iterations.

To assess trends in DF, a serial Monte Carlo model was used based on the DF data in a given core. In each iteration of the model, a random walk was simulated with an initial DF value based on the value observed at the base of the core (when the starting value was selected randomly from among all values observed in the core, the results (data not shown) are effectively the same). Then, values were shifted by adding one first difference, using a first difference value observed between two randomly selected adjacent levels. The process was repeated for all successive levels resulting in a random time series based on the actual first differences observed in the core. If the shift resulted in an impossible negative value of DF, the shift was reversed. Alternative runs with negative values converted to 0 (not shown) yielded comparable outcomes. The process was repeated 10 000 times and the resulting set of 10 000 time series was used to estimate the range of possible trends expected under a random walk. The results were also used to estimate significance of changes in DF across the core. In this approach, the core was divided into two groups of increments (upper and lower parts of the cores) starting from the third increment in the core and then successively shifting the boundary between the lower and upper group to find the dividing point that results in the maximum difference in DF. The difference was measured using *t*-statistic (difference between the mean DFs between the two parts of the core divided by the standard error). The resulting trend was then compared against 95% confidence bands based on the analysis of the 10 000 Monte Carlo series simulating non-directional random trends. In addition, a Spearman rank correlation was computed for DF and core depth (positive correlation indicates an upward decrease in drilling predation and negative correlation indicates an upward increase in DF). The distribution of expected values of Spearman rho was computed based on 10 000 Monte Carlo runs. Monte Carlo simulations and Spearman rank correlations were performed for the total assemblage, gastropods and bivalves at all three stations (except for gastropods at station Po 3 because of a low number of samples with a minimum of 20 individuals; electronic supplementary material, table S3). Statistical analyses were performed with the software package R (v. 4.3.1). All data used in this study and the R code are available at the Dryad Digital Repository [45].

## 3. Results

**Figure 2.**
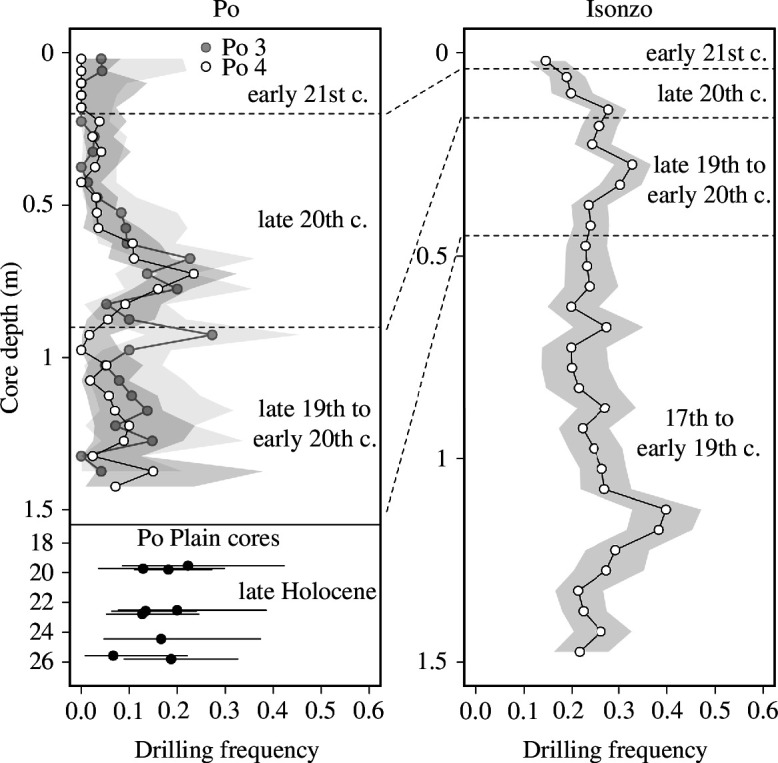
DFs of the total assemblage in the cores decline in the late twentieth century and later in both regions. Note distinct peaks of DFs in the mid-twentieth century in both regions.

The overall DFs in the molluscan assemblages of the Po prodelta exceeded 10% in all but one sample from the middle-late Holocene and fluctuated around 10% in the late nineteenth to early twentieth century (figure 2). The DFs in the Isonzo prodelta were consistently higher, varying between 20% and 30% from the seventeenth to the early twentieth century, rarely exceeding 30%. Despite these differences in DFs, both regions exhibited peaks in DFs in the mid-twentieth century (above 20% in the Po prodelta and above 30% in the Isonzo prodelta), followed by a decrease during the late twentieth and early twenty-first century, with DFs reaching all-time lows in the youngest samples (figures 1*b* and 2). These declines were also observed when bivalves and gastropods were analysed separately (electronic supplementary material, figures S2 and S3). DFs in the total assemblage during the late nineteenth to early twentieth century and the late twentieth century were significantly lower compared with the late Holocene baseline in the Po delta when core increments are grouped into three multi-decadal time bins according to their stratigraphic position (figure 1, table 1*a,b*). Differences between time bins are statistically insignificant in the Isonzo delta (figure 1, table 1*a*).

**Table 1.** Results of statistical tests for significance of differences in drilling frequencies (DFs), abundance of predators and community composition. Bold values indicate significance level <0.01; underlined values indicate significance level <0.05. (*a*) Kruskal–Wallis test for differences of DFs between temporal units in the two regions. (*b*) The *p*-values from the pairwise Wilcoxon test of DFs of temporal units in the two regions were obtained using *a posteriori* Bonferroni correction for multiple comparisons. (*c*) Kruskal–Wallis test for differences in predator abundance between temporal units in the two regions. (*d*) The *p*-values from pairwise Wilcoxon test of predator abundance of temporal units in the Po prodelta, using *a posteriori* Bonferroni correction for multiple comparisons. (*e*) PERMANOVA test for differences in community composition between the two regions and between temporal units within regions. Pseudo-*F*-statistics and probability are listed. (*f*) Distance-based redundancy analysis (RDA) test for the Isonzo and Po prodeltas. The Po prodelta is analysed without the Holocene samples from the Po plain, where depth may not be a good proxy for age. *F*-statistics and probability are listed.

*a*			
	chi-squared	d.f.	*p*‐value
Po prodelta	15.094	2	**0.0005276**
Isonzo prodelta	4.5819	2	0.1012

*b*			
Po prodelta	late Holocene	late nineteenth to early twentieth century	
late nineteenth to early twentieth century	**0.0098**		
late twentieth to early twenty-first century	**0.0012**	0.2567	

*c*			
	chi-squared	d.f.	*p*‐value
Po prodelta	17.713	2	**0.0001425**
Isonzo prodelta	2.6181	2	0.2701

*d*			
Po prodelta	late Holocene	late nineteenth to early twentieth century	
late nineteenth to early twentieth century	**0.01187**		
late twentieth to early twenty-first century	**0.00012**	1	

*e*			
PERMANOVA	*F*-statistics	Pr(>F)	
regions (Po versus Panzano)	32.066	**0.001**	
Po prodelta temporal units	11.207	**0.001**	
Isonzo prodelta temporal units	7.2275	**0.001**	

*f*			
RDA	*F*-statistics	Pr(>F)	
Po	2.1656	0.014	
Panzano	1.5101	0.079	

DFs of the total assemblage exhibit a strong positive autocorrelation among adjacent 5 cm thick levels (lag 1; electronic supplementary material, figures S4–S6). The simulated time series of DFs represent non-directional random walks, which tend to drift towards larger values. The Monte Carlo simulations suggest that the most likely transition from higher to lower DFs occurs around the early 1950s (core depth 15 cm) in Panzano, around 1980s (core depth 50 cm) at Po 3 and at the end of the twentieth century (core depth 22.5 cm) at Po 4 (electronic supplementary material, figures S4–S6). The decrease in DFs of the total assemblage towards the top of the cores is statistically significant for Po 3 and Po 4, but not for Panzano (electronic supplementary material, figure S7). Monte Carlo simulations for DFs of bivalves and gastropods alone show similar trends at the two Po stations, but at Panzano DFs of gastropods also drastically and significantly decrease. At the same time, there is no significant trend for bivalves (electronic supplementary material, figures S7–S12). Also, for the four most abundant species (*V. gibba*, *K. bidentata*, *T. tricarinata*, *T. varicosa*), the results of Fisher’s test consistently indicate that DF decreased through time and, in three out of four cases, the observed difference was statistically significant (*p *< 0.0001 in all three cases; electronic supplementary material, table S4).

**Figure 3.**
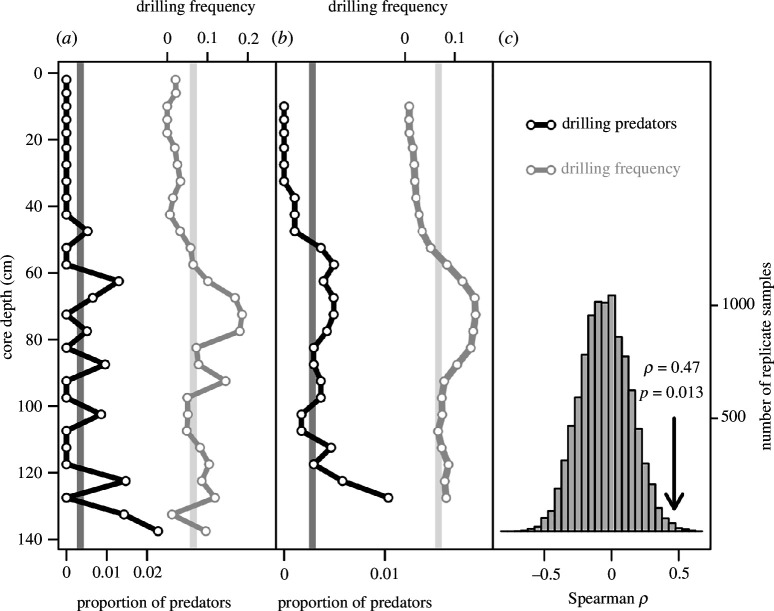
Changes in abundance of drilling predators and DF in Po cores (data combined for all cores). (*a*) Vertical (temporal) changes in the relative abundance of drilling predators and DF in total assemblage as a function of depth in the core. Vertical lines represent the mean relative predator abundance and mean DF for data pooled across all levels in the cores. (*b*) A smoothed trend based on moving average computed as means of successive sets of five adjacent depth levels (core depth based on the middle of the five observations). Vertical lines are the same as in plot *a*. (*c*) Resampling distribution of Spearman rho values for rank correlation between the relative abundance of predators and depth in core. The vertical arrow indicates the location of the observed rho value.

**Figure 4.**
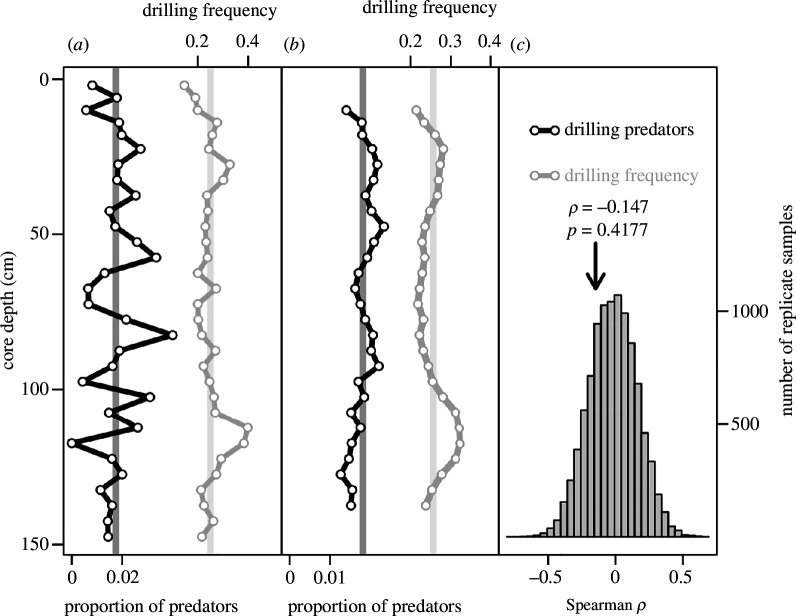
Changes in abundance of drilling predators and DF in Panzano cores (data combined for all cores). (*a*) Vertical (temporal) changes in the relative abundance of drilling predators and DF in total assemblage as a function of depth in the core. Vertical lines represent the mean relative predator abundance and mean DF for data pooled across all levels in the cores. (*b*) A smoothed trend based on moving average computed as means of successive sets of five adjacent depth levels (core depth based on the middle of the five observations). Vertical lines are the same as in plot *a*. (*c*) Resampling distribution of Spearman rho values for rank correlation between the relative abundance of predators and depth in core. The vertical arrow indicates the location of the observed rho value.

Drilling predators (naticid and muricid gastropods) constitute only 0.3% and 0.2% of the total molluscan assemblage at the Panzano and Po stations, respectively (figures 3 and 4, electronic supplementary material, figure S13). A noticeable decline in their relative abundance can be detected in the Po cores (figure 3*a*) and becomes more noticeable when the trend is smoothed using a moving average with a step of 5 (figure 3*b*). Conversely, no decline in drilling predators is discernible at Panzano (figure 4*a*,*b*). The upward decline of drilling predators is statistically significant at the Po stations when measured as rank correlation between core depth and relative abundance of predators (rho = 0.47), as indicated by a Monte Carlo simulation of a null model that assumes constant relative abundance of predators (figure 3*c*), with the observed trend reflecting sampling effects only. The decline in DF in Panzano cores is not distinguishable from a Monte Carlo null model (figure 4*c*). DFs and abundance of predators decline in parallel at both prodelta successions (figure 1*b,c*, electronic supplementary material, figure S14).

**Figure 5.**
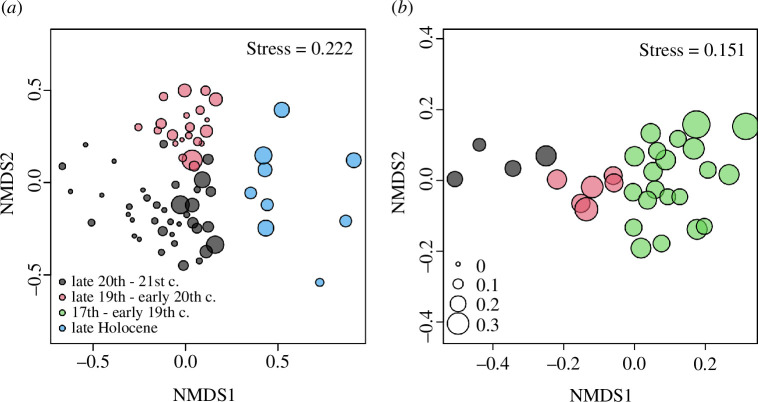
Temporal change in the species composition of molluscan communities, visualized by NMDS, is associated with temporally decreasing DF for (*a*) the Po prodelta and (*b*) the Isonzo prodelta. Size of circles is proportional to DFs.

**Figure 6.**
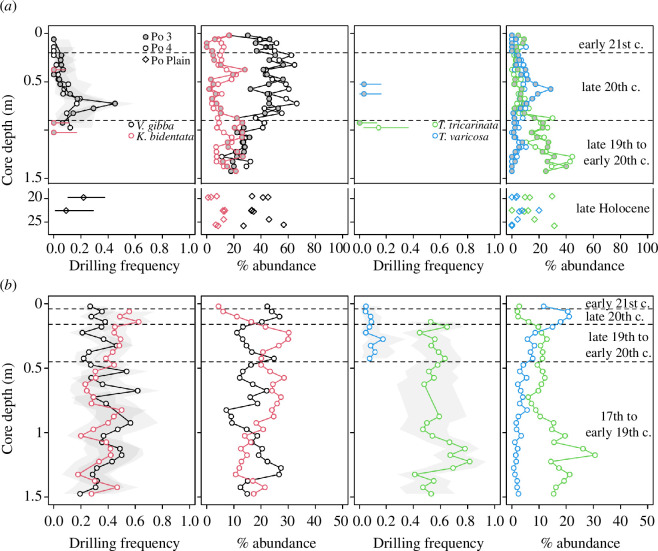
Relative abundance and DFs of the four most abundant prey species in (*a*) the Po prodelta and (*b*) the Isonzo prodelta.

Molluscan assemblages differ between the two regions (electronic supplementary material, figure S15), but show significant temporal changes in composition accompanied by decreasing DFs in both prodelta successions (table 1*e*). However, the correlation between DF and multivariate assemblage composition is significant only in the Po prodelta (figure 5, table 1*f*). The changes in relative abundances of the four most abundant species (representing 37–85% of individuals per sample) are related to species-level trends in DFs, highlighting the link between compositional shifts and overall DF. In the Po prodelta, the strong increase in the relative abundance of the opportunistic bivalve *V. gibba* since the mid-twentieth century is paralleled by the strong decrease of the suspension-feeding gastropod *T. tricarinata* towards the present. The distinct increase in abundance of *V. gibba* coincides with a decrease in its DF, while *T. tricarinata* is never abundant enough in the Po prodelta to confidently evaluate temporal trends in its species-level DF (figure 6*a*). Given that *V. gibba* dominated the molluscan assemblages in that area, the overall DFs followed the trends observed in this species. In the Isonzo prodelta, the relative abundance of the commensal bivalve *K. bidentata* increased steadily until the late nineteenth–early twentieth century and declined rapidly during the twentieth century. The abundance of the scavenger *T. varicosa* shows an opposite pattern with a strong increase during that time, while *T. tricarinata* decreases in relative abundance since the seventeenth century, with a drastic drop during the late twentieth century (figure 6*b*). The trends in the relative abundance of *K. bidentata*, *T. tricarinata* and *T. varicosa* in the Isonzo prodelta affect the DFs of the total assemblage: *K. bidentata* and *T. tricarinata* are frequently drilled and disappear from the community at the expense of *T. varicosa*, which is rarely drilled (figure 6*b*). *V. gibba* in the Isonzo prodelta, however, shows a strong increase in relative abundance since the early twentieth century, which comes along with a slight decrease in DFs (figure 6*b*). Therefore, the community changes in the Isonzo prodelta have only a minor impact on the overall decreasing DFs of the total assemblage (figure 2, table 1*f*).

## 4. Discussion

Predation is one of the key factors shaping marine ecosystems today [45–48]. However, the rarity or lack of baseline data prohibits assessing how predator–prey interactions may have changed under increasing anthropogenic impact on marine ecosystems (e.g. [13]). A longer term perspective, incorporating data from the Holocene fossil record is necessary to answer this question (e.g. [49]). At a regional scale and using sediment cores from areas with low sedimentation rates, DFs in the NAS were significantly lower during the early phase of the Holocene sea-level rise (approximately 10–20% per increment) than during the sea-level highstand over the last few 1000 years (approximately 20–30% per increment). DF largely increases with increasing clay content of sediments and other environmental variables correlated with water depth (organic enrichment and hydrodynamic energy) and with increasing predation pressure (as reflected in the relative abundance of naticid and muricid gastropods) [18]. However, it remains unclear how the approximately twentieth-century compositional replacement of epifaunal grazers, carnivores and herbivores by infaunal opportunistic detritus and suspension feeders [31] influenced predator–prey interactions. We demonstrate here that this mid-twentieth-century compositional shift is associated with a decline in DFs. This decline coincides with a decline in abundance of predatory gastropods in the Po prodelta but not in the Isonzo prodelta. The decline is directly preceded by distinct peaks in predation frequency (figure 2) coinciding with the onset of eutrophication [26,50]. Depending on the number of trophic levels, primary productivity driven by eutrophication can increase the abundance of predators and predator–prey interactions [51,52]. Over millennial time scales, an increase in organic enrichment of sediments (covarying with a decline in grain size) during the Holocene also had a positive effect on DFs in the NAS [18]. However, the positive effects of enhanced food supply on macrobenthic communities were apparently overwhelmed by the more frequent occurrence of seasonal hypoxia since the 1960s [26], which resulted in a loss of formerly abundant, hypoxia-sensitive species such as *T. tricarinata* [35]. A similarly complex relationship between the intensity of drilling predation and primary productivity, modulated by the oxygen availability, was documented in bivalve assemblages sampled along the northern Gulf of Mexico: DFs increased with net primary productivity but the development of hypoxic conditions in the most productive areas adjacent to the Mississippi River apparently limited the activity of drilling predators [53]. Although a decline in salinity might also decrease the abundance of drilling gastropods, salinity did not markedly increase or decrease in the northern Adriatic Sea between 1911 and 1982 and thus does not account for the observed decline [26,54].

Changes in assemblage-level DFs are not only dependent on the identity and abundance of the predators but also on the varying relative abundances of prey species and differences in their susceptibility to drilling predation [55]. Our data suggest that both mechanisms were likely responsible for the late twentieth-century decrease in DFs observed in the NAS. The collapse of predator–prey interactions in the Po prodelta was driven by the complete loss of predatory gastropods in the late twentieth and early twenty-first century (figure 1*c*), while a prominent prey species, *V. gibba*, remained abundant. Conversely, the decrease in predator abundance in the late twentieth and early twenty-first century is small and the decrease of DFs in the Isonzo prodelta is only significant for gastropods (electronic supplementary material, figure S11). The decrease in DFs in the Isonzo prodelta coincides with the replacement of the preferentially drilled species (*K. bidentata* and *T. tricarinata*) by species that are only moderately affected by drilling predation such as *T. varicosa*. As the relative abundance of predators did not decrease, it is possible that they may have switched to a non-drilling predatory strategy [56]. Trends in the relative abundance of predators can also mask decline in their absolute abundance, if the population density of other mollusc species declined in the twentieth century as well. However, Panzano assemblages are time-averaged to centuries rather than to decades (i.e. their faunal composition is more condensed) and thus have smaller potential to record any decline in drilling predators occurring at decadal scales during the twentieth century. Our results could be biased by differences in transportability and thus accumulation of drilled and undrilled shells [57], preferential destruction of undrilled shells by crushing predators like crabs or fish [58], and preferential loss of drilled shells by post-mortem breakage due to reduced strength [59]. However, post-mortem transport of shells was not observed in the studied low-energy muddy environments of the NAS and taphonomic assessment of the fossil assemblages gives no evidence of preferential breakage of drilled shells. Moreover, given the stable water depths at Po and Panzano stations during the late Holocene, these potential biases should be similar for all samples and therefore cannot affect the trend of declining DFs. Biased preservation of drilled shells due to the activity of burrowers is also unlikely. In the Po cores, DF declines in tandem with a decrease in bioturbation intensity related to the onset of frequent hypoxia [35] and it remains low when burrowing increases again in the top of the cores following the reduction in the intensity of hypoxic events in the twenty-first century [29]. Also, the formerly and newly common prey taxa at Panzano have similar preservation potential and belong to families with an excellent Cenozoic fossil record [60].

Ecological release refers to the response of populations to the relaxation of selection acting on some ecologically important traits such as body size and abundance, providing ecological opportunity for an increase in population biomass of prey [61]. The increase in eutrophication and frequency of hypoxia since the mid-twentieth century could produce an ecological opportunity for the hypoxia-tolerant species *V. gibba* [62–65] by removing its competitors and predators, leading to a marked increase in its shell size and relative abundance [25]. We support this hypothesis, showing that twentieth-century ecosystem changes are associated with a decline in the abundance of the hypoxia-sensitive *T. tricarinata* (figure 6) [66], a decline in the abundance of drilling gastropods (figure 1*c*, electronic supplementary material, figure S15) and a decline in DFs in the total assemblage (figure 1*b*).

Although surface death assemblages are valuable sources of historical ecological data [67], in areas with high sedimentation rates only historical layers below the mixed layer can serve as an unequivocal pre-impact ecological baseline [68]. Whereas many studies reconstructed past community states and Anthropocene impacts from sediment cores (e.g. [69–71]), only a few were evaluated for temporal changes in biotic interactions through the Holocene, and those previous analyses focused on parasite–host interactions rather than predation [49,72–74]. Although studies on marine invertebrate communities enabled the discovery of fundamental processes and mechanisms driving community structure and function (e.g. [75–77]), anthropogenic impacts on predator–prey interactions were documented for top vertebrate predators only (e.g. [4]). Our results from the NAS suggest that, although the region has been affected by humans at least since the Roman times [21], it was not until the twentieth century that human activities began to modify invertebrate predator–prey interactions strongly. This time lag is likely related to a shift in the nature and intensity of anthropogenic impacts with significant changes to water quality of the Adriatic Sea starting only in the nineteenth century [21]. The late twentieth-century increase in eutrophication-driven hypoxia resulted in a strong decline in the frequency of predator–prey interactions at lower trophic levels due to a decrease in predator abundance, a turnover towards less-preferred prey organisms and a size increase in a dominant prey species. These changes were accompanied by the strong depletion of marine resources in the study area, most notably large predators and consumers since the nineteenth century [21,30] and the basin-wide infaunalization of benthic soft-bottom communities driven by anthropogenic habitat degradation in the twentieth century [31]. All these trends indicate a strong simplification of the food web in the NAS.

## Data Availability

All data used in this study and the R code are available at the Dryad Digital Repository [45].
